# Mechanisms of immunogenicity in colorectal cancer

**DOI:** 10.1002/bjs.11204

**Published:** 2019-06-19

**Authors:** T O Sillo, A D Beggs, D G Morton, G Middleton

**Affiliations:** Institute of Immunology and Immunotherapy, College of Medical and Dental Sciences, University of Birmingham, Birmingham, UK; Institute of Cancer and Genomic Sciences, College of Medical and Dental Sciences, University of Birmingham, Birmingham, UK; Academic Department of Surgery, College of Medical and Dental Sciences, Queen Elizabeth Hospital, Birmingham, UK

## Abstract

**Background:**

The immune response in cancer is increasingly understood to be important in determining clinical outcomes, including responses to cancer therapies. New insights into the mechanisms underpinning the immune microenvironment in colorectal cancer are helping to develop the role of immunotherapy and suggest targeted approaches to the management of colorectal cancer at all disease stages.

**Method:**

A literature search was performed in PubMed, MEDLINE and Cochrane Library databases to identify relevant articles. This narrative review discusses the current understanding of the contributors to immunogenicity in colorectal cancer and potential applications for targeted therapies.

**Results:**

Responsiveness to immunotherapy in colorectal cancer is non-uniform. Several factors, both germline and tumour-related, are potential determinants of immunogenicity in colorectal cancer. Current approaches target tumours with high immunogenicity driven by mutations in DNA mismatch repair genes. Recent work suggests a role for therapies that boost the immune response in tumours with low immunogenicity.

**Conclusion:**

With the development of promising therapies to boost the innate immune response, there is significant potential for the expansion of the role of immunotherapy as an adjuvant to surgical treatment in colorectal cancer.

## Graphical Abstract

This narrative review highlights current understanding of the mechanisms of immunogenicity in colorectal cancer. The roles of germline, somatic and epigenetic factors in determining the immune environment in colorectal cancer are considered, and the significant potential for targeted immune therapies to improve patient outcomes is discussed.

**Graphical Abstract:**
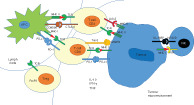

Role for the future

## Resumen

**Antecedentes:**

La respuesta inmune en el cáncer se considera cada vez más importante por su influencia sobre los resultados clínicos, incluidas las respuestas a las diferentes modalidades de tratamiento. Los nuevos conocimientos sobre los mecanismos implicados en el microambiente inmunitario en el cáncer colorrectal están ayudando a definir el papel de la inmunoterapia y el desarrollo de terapias dirigidas para el tratamiento del cáncer colorrectal en todos los estadios de la enfermedad.

**Métodos:**

Se realizó una búsqueda bibliográfica en las bases de datos PubMed, Medline y Cochrane para identificar artículos relevantes. Esta revisión descriptiva discute la comprensión actual de los factores que contribuyen a la inmunogenicidad en el cáncer colorrectal y las posibles aplicaciones en terapias dirigidas.

**Resultados:**

La capacidad de respuesta a la inmunoterapia en el cáncer colorrectal no es uniforme. Varios factores, tanto relacionados con la línea germinal, como con el tumor son determinantes potenciales de la inmunogenicidad en el cáncer colorrectal. Los estudios actuales están dirigidos a tumores con alta inmunogenicidad provocada por mutaciones en los genes de reparación de apareamientos erróneos en el ADN. Trabajos recientes sugieren un papel para los tratamientos que estimulan la respuesta inmune en tumores con baja inmunogenicidad.

**Conclusión:**

Con el desarrollo de tratamientos prometedores para estimular la respuesta inmune innata, existe un potencial significativo para la expansión del papel de la inmunoterapia como adyuvante del tratamiento quirúrgico en el cáncer colorrectal.

## Introduction

The tumour microenvironment in colorectal cancer is influenced by somatic mutational and epigenetic events that occur during tumour development, as well as by the host immune system, which exerts negative selection pressures on tumour cells, by recognition of tumour antigens as non-self^1^. Immune checkpoints are a series of innate and adaptive regulatory mechanisms to modulate immune activity and promote tolerance to self-antigens. These can be upregulated in tumours to drive resistance to immune cell-mediated destruction^2^^,^^3^. Immunotherapy has been most successful in targeting and blocking these immune checkpoints, leading to effective antitumour responses in some cancers^4^.

The emergence of immunotherapy has transformed the treatment landscape of some cancers, most notably cutaneous melanoma^5^^,^^6^ and non-small cell lung cancer (NSCLC)^7^^,^^8^. So far, the role of immunotherapy in colorectal cancer been limited to the 3–4 per cent of patients with metastatic disease whose tumours demonstrated microsatellite instability (MSI)^9^, due to germline, somatic or epigenetic inactivation of DNA mismatch repair (MMR) genes^10^. However, its role could be expanded significantly by drawing on an understanding of the immunogenomic drivers of the response in the tumour environment.

This review explores current understanding of the relative contributions of innate immune genomic mechanisms and somatic mutations to the immune environment in colorectal cancer, with the implications for potential expansion of the roles of immunotherapy and other targeted therapies in the management of colorectal cancer at all disease stages.

## Methods

### Search strategy

A literature search was conducted using the PubMed, MEDLINE and Cochrane Library databases, as well as reference lists from appropriate papers. The goal was to provide an overview of published research in the field of colorectal cancer genomics and immunology, with a particular focus on advances since the launch of the genomics era after completion of the Human Genome Project^11^. The following keywords were used to perform flexible searches within these databases: ‘immunotherapy’, ‘colorectal’ AND ‘cancer’, ‘mutation’, ‘immunity’ and ‘immunologic adjuvants’. Only papers published in English were included.

### Structure

An overview of the role of MSI in colorectal cancer in delineating clinical outcomes and the response to immunotherapy is presented, followed by an in-depth consideration of current understanding of the determinants of the colorectal tumour environment, including tumour mutational factors, inherited germline determinants and the potential role of the gut microbiome. The implications of immune heterogeneity in colorectal cancer and clinical applications for immunotherapeutic approaches are considered. There is a strong argument for routine testing and treatment of patients with colorectal cancer based primarily on immunogenomic rather than histopathological markers.

## Microsatellite instability in colorectal cancer

Approximately 15 per cent of patients with colorectal cancer have tumours that demonstrate MSI, secondary to deficient MMR (dMMR). MSI – high (MSI-H) tumours are characterized by a high mutational burden and the generation of large numbers of neoantigens, which trigger powerful anticancer host immune responses^12–14^. In contrast, the 85 per cent of colorectal cancers that develop owing to chromosomal instability, termed microsatellite stable (MSS)^15^, has a much lower mutational burden and smaller numbers of neoantigens.

More than two variants of MSI-H colorectal cancer have been demonstrated^10^^,^^16^. Hereditary non-polyposis colonic cancer or Lynch syndrome is found in 3 per cent of colorectal cancers. It is caused by an inactivating germline mutation of one or more of the DNA MMR genes (*MLH1, MSH2, MSH6* and *PMS2*), with a second hit from a sporadic mutation, loss of heterozygosity or epigenetic silencing of a second MMR gene^10^. These patients have a 50–70 per cent lifetime risk of colorectal cancer, as well as significant lifetime risks of endometrial cancer (in women), and other intestinal and urothelial cancers^17^. More commonly, MSI-H tumours have no underlying germline mutations, and arise as a consequence of epigenetic silencing of the MMR gene *MLH1* by hypermethylation of its promoter region^18^. Sporadic MSI-H colorectal cancer is frequently associated with the v-raf murine sarcoma viral oncogene homolog B1 *(BRAF)* V600E mutation, through its association with the CpG island methylator phenotype.

BRAF is a downstream molecule in the Rat sarcoma protein (Ras)–mitogen-associated protein kinase (MAPK) signalling pathway, which is critical for cell survival and proliferation^19^. *BRAF* mutations are present in both sporadic MSI-H and MSS colorectal cancers but mostly absent in Lynch syndrome, and so the presence of a *BRAF* mutation, in conjunction with *MLH1* methylation analysis, reliably distinguishes between sporadic MSI-H colorectal cancer and Lynch syndrome^20^. A third variant, Lynch-like syndrome, is less well characterized. Lynch-like colorectal tumours have no germline MMR gene mutations or hypermethylation of the *MLH1* promoter^21^, suggesting other unknown somatic mutations within MMR genes as the cause of MSI^10^. The revised Bethesda guidelines for Lynch syndrome diagnosis^22^ take into account both clinical information (including diagnosis at a young age and strong family history) and assessment of MSI status by immunohistochemistry or genomic analysis.

MSI-H colorectal cancers have clinicopathological features distinct from those of MSS tumours, including an increased incidence in female patients, more proximal colonic location, high lymphocyte infiltration levels and lower incidence of metastasis, with better clinical prognosis at stage I–III^13^^,^^23^. A nationwide study^24^ of 6692 patients by the Danish Colorectal Cancer Group revealed a reduced risk of synchronous metastases in patients with dMMR colorectal cancer (8·0 *versus* 15·8 per cent; odds ratio 0·54). There was also an inverse association between dMMR status and lymph node metastasis and venous invasion. However, in metastatic (stage IV) disease, MSI appears to confer a worse prognosis^25^.

MMR loss is associated with the rapid accumulation of mutations. Timmermann and colleagues^26^ performed whole-exome sequencing (WES) of MSI and MSS colorectal cancers in two patients, and found 1304 somatic mutations in the MSI tumour compared to 198 in the MSS lesion. In addition to base substitutions, large numbers of insertions and deletions occur^20^. They may lead to frameshifts which, if occurring in tumour suppressor genes, can drive tumorigenesis. High mutation rates generate large numbers of new peptides, termed neoantigens, which are not recognized as self and thus are strongly immunogenic. Neoantigens contribute to a better prognosis in MSI colorectal cancer owing to the increased infiltration of effector cells (primarily effector T cells^27^) into the tumour environment^13^^,^^23^.

Other mechanisms may also contribute towards immunogenicity in MSI-H colorectal cancer. Constitutive activation of the viral response cyclic guanosine–adenosine 3′,5′-cyclic monophosphate synthase–stimulator of interferon genes (cGAS-STING) pathway, with associated T cell infiltration, occurs in DNA damage response-deficient breast cancers^28^. cGAS is activated by DNA damage and localizes to micronuclei that form during tumorigenesis^29^. This triggers a proinflammatory response. Deficiency in the MMR protein MLH-1 is associated with deficient DNA double-strand break repair and increased micronuclei formation^30^, which may also trigger the cGAS-activated inflammatory response.

Current immunotherapeutic approaches serve primarily to block immune checkpoints, boosting immune-mediated tumour destruction^31^. Patients with dMMR metastatic colorectal cancer have been shown to have significant clinical responses to immunotherapy with antiprogrammed cell death 1 (PD-1)/antiprogrammed cell death ligand 1 (PD-L1) treatment in phase II trials^21^, in stark contrast to those in the MSS colorectal cancer subgroup where there was no objective response to immunotherapy^32^. Yarchoan and co-workers^33^ demonstrated a strong correlation between tumour somatic mutation frequency (and therefore neoantigen burden) and the response to immunotherapy across a range of human cancer subtypes.

However, MSI status and neoantigen burden do not sufficiently explain the variability in the colorectal tumour environment. About 20 per cent of patients in the MSS colorectal cancer subgroup develop an immunogenomic signature similar to that in MSI-H colorectal cancer, despite low mutational burden^34^. There is evidence that activating mutations in the Ras–MAPK pathway are associated with lower expression of this immune gene cluster and immune pathway downregulation^35–37^. In addition, lymphocytic infiltration, particularly of effector and memory T cells into the tumour, a key indicator of prognosis in colorectal cancer^27^^,^^38^, appears to be independent of MSI status^39^.

## Colorectal cancer tumour microenvironment

Various mechanisms lead to immunosuppression in colorectal cancer. Recruitment of immunoregulatory cells^40^, upregulation of inhibitory molecules (including myeloid-derived suppressor cells (MDSCs), T regulatory (Treg) cells, type 2 macrophages and other cancer-associated cell types^2^^,^^41–43^) and downregulation of antigen presentation represent methods of immune evasion^44^. The alteration of metabolic pathways to favour glycolysis, even in the presence of sufficient oxygen, is termed the Warburg effect^45^. This, along with the upregulation of anabolic pathways that favour rapid tumour cell survival and proliferation, often leads to the generation of an environment that is hostile to T cells owing to increased acidity, low oxygen levels, competition for nutrients and the generation of waste substrates^44^^,^^46^. In this context, T cell exhaustion occurs, defined as the presence of T cells with decreased cytokine expression and effector function^47^^,^^48^.

Activated T cells express inhibitory co-receptors, termed immune checkpoints. The best characterized include PD-1, cytotoxic T lymphocyte-associated protein 4, lymphocyte-activation gene 3, T cell immunoglobulin mucin 3 (Tim-3) and killer immunoglobulin-like receptors. When they bind to ligands present on antigen-presenting cells and other cells in the immune environment, they downgrade the inflammatory response^42^. This serves as an innate mechanism to maintain self-tolerance and limit immune-mediated tissue damage.

**Fig. 1:**
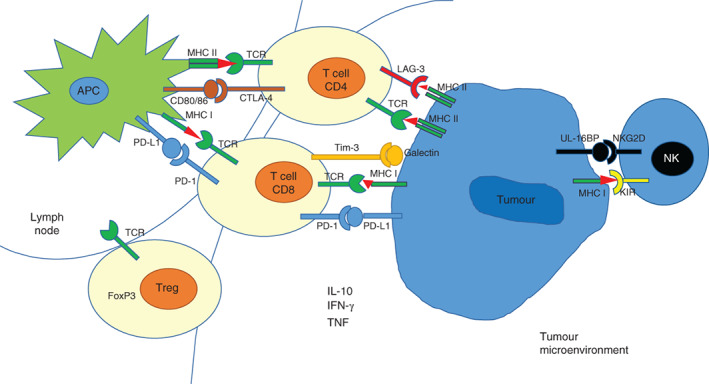
Immune regulatory pathways in the tumour microenvironment

Adapted from Postow *et al*.^42^ and Wachowska and colleagues^43^. APC, antigen-presenting cell; CTLA-4, cytotoxic T lymphocyte-associated protein 4; FoxP3, forkhead box P3; IFN-γ, interferon γ; IL-10, interleukin 10; KIR, killer immunoglobulin-like receptor; LAG-3, lymphocyte activation gene 3; NK, natural killer cell; NKG2D, natural killer G2D receptor; PD-1, programmed cell death 1 protein; PD-L1, programmed cell death 1 ligand; TCR, T cell receptor complex; Tim-3, T cell immunoglobulin mucin 3; TNF, tumour necrosis factor; Treg, regulatory T cell; UL16BP, UL16-binding proteins.

Selective upregulation of these immune checkpoints is often present in MSI-H tumours (*Fig. 1*). This may explain why MSI-H tumours are not eliminated naturally despite high immune activation, and why checkpoint blockade is effective in these tumours. Tumour infiltrating lymphocytes (TILs) in MSI-H colorectal cancer express high levels of PD-1, which is absent in MSS colorectal cancer. However, corresponding expression of immune checkpoint ligands is often absent on tumour cells, and found to be present on a population of infiltrating myeloid cells^31^. Immature populations of myeloid cells (MDSCs) are present in most tumours and are induced in the presence of cancer cells^49^. The upregulation of PD-L1 on these cells suggests a direct interaction with T cells. They also appear to increase toxic cell metabolite production and induce Treg activity, which further suppress effector cell activity^49^. Tim-3, which blocks T helper responses, is often upregulated on exhausted CD4+ and CD8+ TILs in colorectal cancer in combination with PD-1^50^. This correlates with regional metastases and poorer prognoses in both colorectal cancer and other solid tumours ^51^^,^^52^.

Clinicopathological data strongly suggest that effector T cells in the tumour microenvironment are key determinants of outcomes. Patients with large TIL numbers have improved survival at all disease stages. This prognostication is superior to that of the UICC TNM classification by disease stage^53^. Galon *et al*.^54^^,^^55^ developed an immunocytochemical score for the colorectal cancer immune microenvironment, the Immunoscore® (HalioDx, Marseille, France). It is based on the finding that the infiltration of cytotoxic (CD8+) and memory (CD45RO+) T cells is associated with improved prognosis. The densities of CD45RO+ and CD8+ cells in the centre of the tumour (CT) and invasive margin (IM) are used to stratify patients into distinct populations with significantly different clinical outcomes at all disease stages^53^. In multivariable analysis, after adjusting for tumour category, differentiation, lymph node invasion and other molecular biomarkers including microsatellite status and *BRAF* mutation status, T cell infiltration (CD3_CT_/CD3_IM_) remained an independent prognostic factor for disease-free survival (DFS) and overall survival (OS).

The Immunoscore® was independently validated by the Society for the Immunotherapy of Cancer worldwide consortium study^56^^,^^57^, in 2681 patients from 14 centres across 13 countries. Similarly, in a study^39^ across three cohorts, including 270 colorectal cancer and 3659 pan-cancer samples from the Cancer Genome Atlas, the Immunoscore® was a better predictor of disease-specific survival, DFS and OS than microsatellite status.

Other immune cells contribute to the immune environment. Natural killer (NK) cells are critical in the innate immune response and have spontaneous cytotoxic effects against aberrant cells. There appears to be a decrease in NK cell activity in patients with colorectal cancer compared with healthy controls^58^. In addition, infiltration of NK cells into colorectal tumours appears to be associated with better clinical outcomes. In metastatic disease, both a high proportion of NK cells in peripheral blood and increased NK cytotoxicity are associated with increased responses to chemotherapy and longer survival^59^^,^^60^. However, their interactions with T cells and prognostic significance are not yet understood. Dendritic cells are key antigen-presenting cells with a central role in the initiation and regulation of adaptive immunity. They prime antitumour responses by presenting tumour antigens to T cells and through interactions with other effector cells. Impairment in dendritic cell function occurs in many cancer models and represents a mechanism of immune escape^61^. They also express immune checkpoint ligands, including PD-L1 and CD80/86^62^.

Another mechanism for immunosuppression is the loss of MHC class I and II proteins from cell surfaces. They are required for antigen presentation to T cells and other effector cells. Class I loss is frequent in MSI-H colorectal cancer (60 per cent of cases *versus* 17 per cent of MSS colorectal cancers^63^). Class II expression is more nuanced. It is expressed in up to 50 per cent of colorectal cancers. Subsequent loss of class II expression correlates with reduced TIL density and increased incidence of regional metastases^64^. In melanoma, class II-negative patients had lower objective response and survival rates when treated with anti-PD-1/PD-L1 immunotherapy^65^. Mouse cancer models suggest that induction of class II expression in colorectal cancer may improve tumour immunogenicity. Transfection of the master transcriptional activator of class II (CIITA) into poorly immunogenic class II-negative adenocarcinoma cell lines resulted in these cells developing robust antigen-processing function, with massive infiltration by both CD4+ and CD8+ T cells, and tumour rejection occurred when the CIITA-transfected cell lines were infused into mice^66^^,^^67^.

Although immune cell quantification methods such as the Immunoscore® give a phenotypic output of the colorectal cancer immune environment, the relative contributions of germline, somatic and epigenetic variations in the immune signature to this microenvironment have not been determined. A key question is what drives the presence of large numbers of TIL in some tumours and not others. It is clear that somatic mutational factors alone are not sufficient to explain this variability.

### Implications of immune heterogeneity in colorectal cancer

Colorectal cancer can be divided into four consensus molecular subtypes (CMS), each with distinguishing pathological features^68^. The MSI-H group represents CMS1, showing hypermutation and strong immune activation; CMS2 (canonical) shows chromosomal instability with marked Wnt and myc signalling; CMS3 (epithelial) shows metabolic dysregulation; and CMS4 (mesenchymal) shows prominent transforming growth factor β activation, stromal invasion and angiogenesis. This subtype demonstrates strong immune cell infiltration.

In a recent study^34^ using a T helper-1 centric immune metagene as a marker of the immune contexture, 20 per cent of patients in the MSS colorectal cancer subgroup had an immune signature very similar to that of MSI-H colorectal cancer, despite small numbers of mutations and fewer neoantigens^34^. This group segregated to the CMS4 subtype. The Kirsten ras sarcoma oncogene (*KRAS*) mutation, especially in the CMS2 and 3 subtypes, is associated with downregulation of immune pathways and reduced immune cell infiltration^35^. *KRAS* mutation, apart from predicting non-response to antiepidermal growth factor receptor (EGFR) chemotherapy, is independently associated with a worse prognosis in colorectal cancer^69^.

KRAS and BRAF are downstream molecules in Ras–MAPK signalling, which is a critical mediator of EGFR-induced signalling cascades^70^. Mutations that cause dysregulation and hyperactivation of this pathway^71^ may be implicated in suppression of immunogenicity in colorectal cancer. In a study of triple-negative breast cancer, which is associated with early metastasis and a worse prognosis than other variants, alterations in Ras–MAPK signalling correlated with low TIL numbers, which correlated with worse recurrence-free survival. Using *in vitro* and *in vivo* mouse-derived breast cancer cell lines, inhibition of MAPK kinase (MEK), another downstream molecule in the MAPK signalling cascade, upregulated both MHC class I and II and PD-L1. Combined PD-1/PD-L1 and MEK inhibition enhanced antitumour immune responses^37^.

Lochhead and colleagues^25^ undertook a prospective observational analysis of the impact of *BRAF* mutation status and MSI on 5-year cancer-specific survival in 1253 patients with colorectal cancer. Patients with the MSI-H/*BRAF* wild-type subtype had the highest survival rate (79 per cent), whereas those with the MSS/*BRAF* mutant subtype (46 per cent) had the poorest survival. MSI-H/*BRAF* mutant and MSS/*BRAF* wild-type subtypes had intermediate values (73 and 65 per cent respectively) with no direct interaction between MSI and *BRAF* status. A pooled analysis^72^ of four phase III studies of first-line treatment of metastatic colorectal cancer showed a higher incidence of the *BRAF* mutation in metastatic MSI colorectal cancer. Although the *BRAF* mutation was independently associated with a worse prognosis, subanalysis of the MSI-H and MSS colorectal cancer subgroups established no difference in survival in *BRAF* mutant and *BRAF* wild-type MSI-H colorectal cancers. Metastatic Lynch and Lynch-like colorectal cancers (in which *BRAF* mutations are largely absent) have increased DFS and OS compared with sporadic MSI colorectal cancer, although the typically younger age of patients with Lynch syndrome is a confounding factor^16^.

Large phase III clinical trials^73^^,^^74^ support a combination of BRAF and MEK inhibition in melanoma. Disappointingly, these results were not replicated in combination trials in *BRAF*-mutated colorectal cancer^75^, likely due to the development of escape mechanisms. One possibility is the heterodimerization of BRAF with CRAF, a BRAF isoform, which drives increased Ras–MAPK signalling^76^, and has been noted in the development of resistance and secondary tumours following BRAF inhibition in metastatic melanoma^77^^,^^78^.

### Role of neoantigens in cancer immunotherapy

During tumour evolution, driver mutations, which cause the transformations required for tumorigenesis and tumour propagation, and passively acquired passenger mutations occur. Neoantigens arise as a result of non-synonymous somatic mutations during tumour evolution^79^. They may be clonal (expressed in all tumour cells) or subclonal (expressed in a proportion of tumour cells). Tumours harbouring large numbers of subclonal mutations have a variety of cell populations with different genomic and, therefore, phenotypic signatures^80^. Neoantigen clonality plays a role in determining the likelihood of a durable response to immunotherapy. In a series of NSCLC samples from the Cancer Genome Atlas^80^, patients with tumours with high levels of subclonal mutations (and therefore low neoantigen clonality) had no durable clinical benefit from immunotherapy, irrespective of the neoantigen load. Similarly, in a study^81^ demonstrating the predictive power of mutational burden for response to pembrolizumab, an anti-PD-1 inhibitor, its efficacy was dependent on neoantigen clonality. Tumours with similar neoantigen numbers responded significantly more favourably if neoantigens were clonal than if they were subclonal.

The most potent T cell responses are against neoantigens^82^. As the pattern of mutations is highly variable, and the cancer genome is unique to each individual, identification of neoantigens was challenging initially. With the development of next-generation sequencing and bioinformatics strategies for *in silico* prediction, it is now possible to rapidly identify and filter neoantigens^83–85^. WES of tumour samples allows identification of somatic mutations, which are modelled using a protein prediction algorithm^86^ and fed into an MHC-binding predictor to model the MHC-binding capacity^87–89^. Structural variants (in particular, gene fusions that may also generate neoantigens) are more difficult to identify from WES unless RNA sequencing data are available^90^.

Proposed advantages of targeted cancer immunotherapy include increased efficacy and specificity, resulting in lower toxicity than current treatments. Current approaches involve either boosting the T cell response to tumour neoantigens (adoptive cell transfer and checkpoint blockade are examples of this) or altering the neoantigen landscape to favour the expression of those that are highly immunogenic^90^. Adoptive cell transfer of T cells recognizing certain tumour antigens has been shown to induce tumour regression in some trials, most notably in melanoma^91^. In a clinical trial^92^ in three patients with melanoma, WES was used to identify the highest binding epitope peptides and these patients were vaccinated with autologous dendritic cells that had been pulsed with the top seven highest binding peptides identified from each tumour. This led to an increase in the breadth and diversity of neoantigen-specific T cells from all patients, who were alive with no adverse autoimmune events at the time of reporting. The potential for use in solid tumours, such as breast cancer, is being explored^93^.

Tumour neoantigens are ideal targets for cancer immunotherapy, as they are expressed only in tumour cells and so are less likely to induce either immunological tolerance or toxicity from targeted therapy. However, targeting specific neoantigens may lead to tumour escape via expansion of subclonal populations. It remains uncertain whether cancer vaccination is potent enough to induce remission in solid tumours. Other limiting factors include the significant financial implications inherent in developing personalized treatments, and the possibility of significant adverse reactions. Nevertheless, there are encouraging results from initial studies, and refinements in neoantigen targeting and vaccine delivery are ongoing.

## Applications of immunotherapy in colorectal cancer

Phase I trials of immunotherapy in patients with advanced colorectal cancer showed poor results, with little objective clinical response or improvement in outcomes^94^^,^^95^. However, further studies showed clear differences in those with dMMR/MSI-H colorectal cancer. Le *et al*.^32^ compared outcomes in patients with or without dMMR colorectal cancer who were given pembrolizumab. The immune-related objective response rate (ORR) was 40 per cent and the progression-free survival (PFS) rate 78 per cent in patients with dMMR, compared with 0 and 11 per cent respectively in patients without dMMR. This was associated with a significantly reduced risk of death or disease progression (hazard ratio 0·22) in the dMMR group. High levels of somatic mutations also correlated with improved survival^32^.

Current trials^9^^,^^96^ have not shown significant differences in ORR and disease control in Lynch *versus* non-Lynch-associated tumours. Le and colleagues^96^ observed no significant difference in ORR, determined radiologically and clinically, between Lynch and non-Lynch syndrome-associated MSI-H tumours (46 and 59 per cent respectively; *P* = 0·27). In the Keynote-142 phase II open-label trial^9^ of nivolumab, an anti-PD1 antibody, in patients with metastatic MSI-H colorectal cancer who had been unable to tolerate previous chemotherapy or whose disease had progressed, ORR and disease control rates were similar in Lynch *versus* non-Lynch MSI-H colorectal cancer (33 *versus* 29 per cent, and 70 *versus* 75 per cent, respectively).

Based on data from five single-arm multicohort multicentre trials, in 2017 the US Food and Drug Administration^97^ granted accelerated approval for use of the anti-PD-1 inhibitor pembrolizumab in people with unresectable or MSI-H or dMMR solid tumours. Several ongoing clinical trials are assessing checkpoint blockade agents in colorectal cancer. Patients in these trials all have advanced or metastatic disease (MSI-H and MSS) and have been heavily pretreated^32^^,^^98–103^ (*Table 1*).

**Table 1:** Clinical trials of immunotherapy in colorectal cancer

Reference (trial)	Phase	Regimen	Subgroups	Outcomes	Duration
Le *et al*.^32^	II	Pembrolizumab (PD-1 inhibitor)	dMMR/MSI-H *versus* MSS CRC	Immune-related objective response rate PFS	20 weeks
Overman *et al*.^9^ (CheckMate 142)	II	Nivolumab (PD-1 inhibitor) +/– ipilimumab (CTLA-4 inhibitor)	Metastatic pretreated dMMR/MSI-H CRC	Immune-related objective response rate PFS OS	12 months
Mettu *et al*.^99^ (BACCI)	II	Capecitabine/bevacizumab +/–atezolizumab (PD-L1 inhibitor)	Metastatic CRC	PFS OS	Ongoing
Hoffmann-La Roche^100^ (COTEZO IMblaze370)	III	Cobimetinib + atezolizumab (PD-L1 inhibitor) *versus* atezolizumab monotherapy *versus* regorafenib	Heavily pretreated locally advanced or metastatic CRC (> 95% MSS)	OS PFS	3 years
Diaz *et al*.^101^ (KEYNOTE-177)	III	Pembrolizumab (PD-1 inhibitor) *versus* standard chemotherapy	dMMR/MSI-H stage IV CRC	PFS OS	57 months
Asan Medical Centre^102^ (POLE-M)	III	Standard 5-FU-based adjuvant chemotherapy +/–sequential avelumab (PD-L1 inhibitor)	Resected stage III dMMR/MSI-H or POLE-mutant colonic cancer	DFS	5 years
Sinicrope *et al*.^103^ (ATOMIC, Alliance A021502)	III	Combination chemotherapy +/– atezolizumab (anti-PD-L1) continued as monotherapy for additional 6 months	Resected stage III dMMR/MSI-H colonic carcinomas	DFS OS Adverse events	5 years
Tabernero *et al*.^104^	I	CEA-TCB antibody +/– atezolizumab (anti-PD-L1)	Heavily pretreated metastatic CRC (mainly MSS)	Adverse events Antitumour activity (RECIST version 1.1 criteria^105^) PFS	40 months

PD-1, programmed cell death protein 1; dMMR, deficient mismatch repair; MSI-H, microsatellite instability –high; MSS, microsatellite stable; CRC, colorectal cancer; PFS, progression-free survival; CTLA-4, cytotoxic T lymphocyte-associated protein 4; OS, overall survival; PD-L1, programmed cell death protein ligand 1; POLE-M, mutated DNA polymerase ϵ; 5-FU, 5-fluorouracil; DFS, disease-free survival; CEA-TCB, carcinoembryonic antigen–T cell-bispecific; RECIST, Response Evaluation Criteria in Solid Tumours.

Meta-analysis^106^ of eight clinical trials of immunotherapy with PD-1/PD-L1 blockade has shown that the PD-L1 expression level in tumour samples has neither prognostic nor predictive significance in determining outcomes. These were studies of advanced urothelial and head-and-neck tumours. Similar findings were reported from a study^32^ comparing outcomes after treatment with pembrolizumab in MSI-H and MSS colorectal cancers. PD-L1 expression was detected only in MSI-H tumours, but there was no correlation between PD-L1 levels and PFS or OS.

Another approach is to stimulate immunogenicity within the tumour, for example, by the use of T cell-targeted bispecific antibodies^107^. Bacac and co-workers^108^ assessed carcinoembryonic antigen–T cell-bispecific (CEA-TCB) antibody, which binds simultaneously to CD3 expressed on T cells and CEA, a marker often overexpressed in colorectal cancer. CEA-TCB antibody activity drives T cell proliferation and cytokine release, converting a poorly immunogenic tumour microenvironment into an inflamed one^108^. Thus, CEA-TCB antibody can enhance the effect of immune checkpoint blockade agents, even in MSS tumours. A phase I study^104^ assessing the effect of combination treatment with a novel CEA-TCB antibody (RO6958688) and PD-L1 inhibitor (atezolizumab) in patients with heavily pretreated metastatic colorectal cancer showed increased tumour inflammation and radiological evidence of tumour reduction in patients with both MSI-H and MSS colorectal cancer treated with higher-dose combination therapy (*Table 1*).

Radiotherapy may also stimulate neoantigen generation. Radiation triggers local and systemic immune effects by inducing lethal DNA damage, which increases the visibility of tumour to the host immune environment^109^. The abscopal effect, in which tumour regression occurs at a site distant from the local radiotherapy field, is commonly observed following radiotherapy^110^. Immune cell infiltration into the tumour environment is crucial for this response and is often lacking in the presence of systemic immunosuppression^111^^,^^112^. Case reports have suggested significant clinical benefit in combined immunotherapy and radiotherapy, notably in melanoma^113^. The PACIFIC trial^114^, a phase III randomized trial of the PD-L1 antibody durvalumab as consolidation therapy after radiotherapy in NSCLC, showed significant improvement in PFS with durvalumab compared with placebo (16·8 *versus* 5·6 months). However, the potential for toxicity must be addressed. Combination therapy may generate both tumour-specific and non-tumour-specific antigens, which may induce autoimmune responses^115^. Furthermore, radiotherapy is established in the treatment of rectal cancer^116^, but is not suitable for colonic tumours, which represent 60–70 per cent of the colorectal disease burden^117^^,^^118^.

Currently, the only immunotherapeutic agents licensed for use in advanced colorectal cancer with dMMR target the programmed cell death pathway^119^. There is significant potential to identify subgroups of patients who may respond to specific immunotherapeutic agents targeting other immune cell-driven pathways. In mouse solid tumour models, targeting both Tim-3 and PD-1 leads to greater antitumour responses than targeting either pathway separately^120^. Combined Tim-3 and PD-1 blockade is currently in early-phase clinical trials in solid tumours^121^. Bispecific antibodies show promise in boosting the innate immune response. In addition, the effects of neoantigen clonality in determining the immune microenvironment may provide opportunities to refine the targets employed in cancer vaccination and adoptive cell transfer, potentially making these valuable adjuncts to surgical treatment in colorectal cancer.

## Germline determinants of immunogenicity in colorectal cancer

**Fig. 2:**
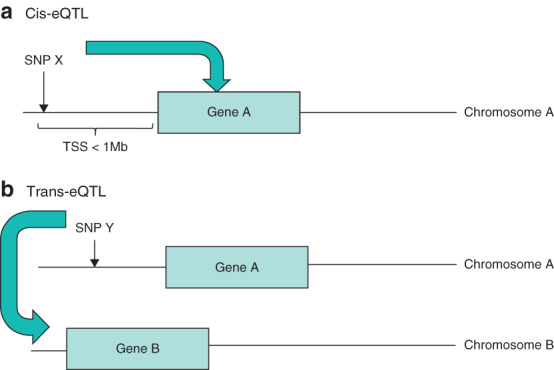
Schematic representation of cis- and trans-expression quantitative trait locus effects on targeted genes

In contrast to exploration of the role of tumour neoantigens in determining immunogenicity in colorectal cancer, the contribution of inherited, germline differences in immune gene expression to the immune landscape is relatively underexplored. A key development has been the expansion of expression quantitative trait locus (eQTL) studies. eQTLs are single-nucleotide polymorphisms (SNPs) usually found in non-coding regions of the genome, which influence gene expression. They may be cis (found in close proximity to the genes they affect) or trans (found at distance from the genes they affect, or even on separate chromosomes)^122–124^ (*Fig. 2*).

**a** Cis-expression quantitative trait locus (eQTL) single-nucleotide polymorphism (SNP) effect on gene A; **b** trans-eQTL effect on gene B on a different chromosome. TSS, transcription start site.

Repositories of eQTL data, such as the Multiple Tissue Human Expression Resource (MuTHER) project^125^ and the Genotype–Tissue Expression Project (GTEx)^126^^,^^127^, have facilitated exploration of the influence of eQTLs in determining the expression of phenotypes of interest, including complex diseases and cancer^128–132^ (*Table 2*). Vogelsang *et al*.^133^ used data from the MuTHER project to identify immune gene eQTLs and correlate these with outcomes in cutaneous melanoma. Of the 382 immunomodulatory genes selected, SNP genotyping of the 50 most significant cis-eQTLs in the MuTHER lymphoblastoid cell line database was performed and correlated with outcome. Two SNPs identified were highly correlated with OS, one affecting interleukin 19 expression and the other BATF3 expression. Landmark-Høyvik and colleagues^134^ showed that the expression of MHC class I and II genes in breast cancer survivors was associated with SNPs in 100 genes. Comparison with a matched healthy cohort revealed specific associations with genes enriched for immune system processes. Although the predictive value of these eQTLs has not yet been explored, the detection of relevant immune genes in patients with colorectal cancer and interrogation of their biological roles will provide further targets for therapy.

**Table 2:** Large-scale human expression quantitative trait locus repositories

Project name	Data repository	eQTL	Tissue subtypes	Sample size
MuTHER	http://www.muther.ac.uk/Data.html	Cis	LCL, skin, adipose	856
GTEx	https://www.gtexportal.org/home/	Cis	Multiple	237
Childhood asthma studies^128^^,^^129^	http://csg.sph.umich.edu/liang/imputation/	Cis and trans	EBVL	2642
International HapMap Project^130^	https://www.ncbi.nlm.nih.gov/geo/query/acc.cgi?acc=GSE6536	Cis and trans	LCL	270
Gilad/Pritchard Group	http://eqtl.uchicago.edu/Home.html	Cis and trans	LCL, liver, brain	
Pickrell Lab	http://gwas-browser.nygenome.org	Cis and trans	Multiple	Combined sources^131^
Geuvadis Project	https://www.ebi.ac.uk/Tools/geuvadis-das/	Cis	LCL	465
Blood eQTL^132^	https://genenetwork.nl/bloodeqtlbrowser/	Cis and trans	Peripheral blood	5311

eQTL, expression quantitative trait locus; MuTHER, Multiple Tissue Human Expression Resource Project; LCL, lymphoblastoid cell lines; GTEx, Genotype–Tissue Expression Project; EBVL, Epstein–Barr virus-transformed cell lines.

## Gut microbiome in colorectal immunogenicity

Interactions between gut microbiota, the evolution of colorectal cancer and responses to therapy are complex. In animal models, specific microbes associated with colonic inflammation can drive carcinogenesis. *Bacteroides fragilis* rapidly induces colitis and colonic tumours in mice heterozygous for the adenomatous polyposis coli gene, with marked downregulation of effector T cell responses and upregulation of Treg responses^135^. In patients with colorectal cancer, there is a large degree of heterogeneity in gut microbiota composition, with differences between faecal and mucosal samples, and between proximal and distal tumours^136^. However, the gut microbiota differ significantly between patients with colorectal cancer and healthy controls^136^. It is uncertain whether these altered microbiota are drivers of carcinogenesis rather than passengers, reflecting the immune responses occurring within the colonic mucosa.

Routy and colleagues^137^^,^^138^ showed that abnormal gut microbiome composition could be responsible for non-response to anti-PD-1 immunotherapy in patients with a range of epithelial cancers, mainly NSCLC and renal cell carcinoma. Systemic antibiotic treatment, which alters the gut microbiome, just before commencing immunotherapy led to worsened PFS and OS than that in a comparable non-treated group. Differences in microbe profiles were noted, with an abundance of *Akkermansia municiphilia* and *Alistipes indistinctus* in responders to immunotherapy. Furthermore, faecal mucosal transplantation (FMT) from responders into germ-free or antibiotic-treated mouse tumour models led to significant antitumour responses, with upregulation of dendritic cell and effector T cell responses. This did not occur with FMT from non-responders^137^. In metastatic melanoma, microbiota in responders to immunotherapy demonstrated an abundance of *Bifidobacterium longum, Collinsella aerofaciens* and *Enterococcus faecium*, whereas non-responders had an abundance of *Ruminococcus obeum* and *Roseburia intestinalis*. A germ-free mouse tumour model also demonstrated similar responses to FMT from responders^139^. The translation of these findings into clinical studies, and into patients with colorectal cancer is an exciting potential avenue of interest.

## Overview

Advances in genetics and cancer immunology have improved understanding of the drivers of immunogenicity in cancer and potential mechanisms for treatment, particularly in cancers that are refractory to current therapies. The role of immunotherapy in colorectal cancer in particular is expanding. Recent guidelines^140^^,^^141^ mandate testing biopsy or resected specimens from all patients with colorectal cancer for Lynch syndrome. This involves a genomic or immunohistochemical screen for MSI, followed by further *BRAF* mutational and *MLH1* hypermethylation analysis to distinguish Lynch from non-Lynch colorectal cancer. The emphasis is on making the diagnosis of Lynch syndrome to facilitate intensive screening to improve clinical outcomes. However, screening is not universal and guidelines often limit this to patients aged less than 70 years^140^^,^^141^. Given the prognostic differences in MSI and MSS colorectal cancer outcomes, and the potential for expansion of the role of immunotherapy in this patient group, this information is critically relevant even in those with sporadic MSI-H colorectal cancer.

For those with MSS colorectal cancer, there are currently no clinically applicable immunogenomic tests to determine the efficacy of immunotherapy. Immunohistochemical markers such as the Immunoscore® and data from genome sequencing have shown the clear potential to identify other equally beneficial markers. In addition, adjuvant methods to boost immunogenicity show significant promise. Although many aspects of these therapies are in their infancy, the potential for the development and application of targeted treatments with greater efficacy and reduced toxicity is attractive. It is anticipated that refined and targeted immune therapies will become part of standard treatment regimens in patients with colorectal cancer at all disease stages.
